# Supplementary material for: Transverse optical gradient force in untethered rotating metaspinners
Source: Light Sci Appl. 2025 Jan 8;14:38. doi: 10.1038/s41377-024-01720-x (PMC11706995; doi:10.1038/s41377-024-01720-x)
Supplement: Supplementary file 1 — Supplementary Information forSupplementary Information for Transverse optical gradient force in untethered rotating metaspinners [file 41377_2024_1720_MOESM1_ESM.docx]

**Supplementary Information for:**

**Transverse optical gradient force in untethered rotating metaspinners**

Einstom Engay^1,#^, Mahdi Shanei^1,#^, Vasilii Mylnikov^1^, Gan Wang^2^, Peter Johansson^3^, Giovanni Volpe^2^, and Mikael Käll^1*^

^1^Department of Physics, Chalmers University of Technology, 412 96 Gothenburg Sweden

^2^Department of Physics, University of Gothenburg, 412 96 Gothenburg, Sweden

^2^School of Science and Technology, Örebro University, 701 82 Örebro, Sweden

#Equal contributions.

*Corresponding author: mikael.kall@chalmers.se

**Supplementary Figures**

**
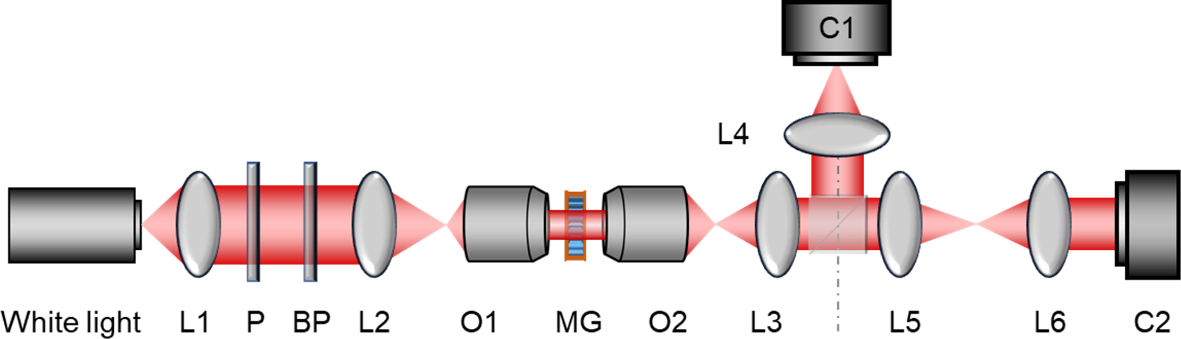
**

**Figure S1. Optical setup for metagrating characterization**. White light is collimated and relayed to the sample plane MG using lenses L1, L2 and microscope objective O1. The beam polarization and center wavelength are set using a polarizer, P and a bandpass filter, BP, with transmission window λ = 1064 $\pm$ 10 nm. The metagrating is imaged onto camera 2 (C2, Thorlabs Zelux) using a 100x oil-immersion objective O2 with NA = 1.49 (Nikon) and a series of relay lenses (L3, L5 and L6). A beam splitter, a Fourier transforming lens (L4) and second camera 1 (C1, Thorlabs Zelux) are employed to record the diffraction pattern in the Fourier/*k*-space.


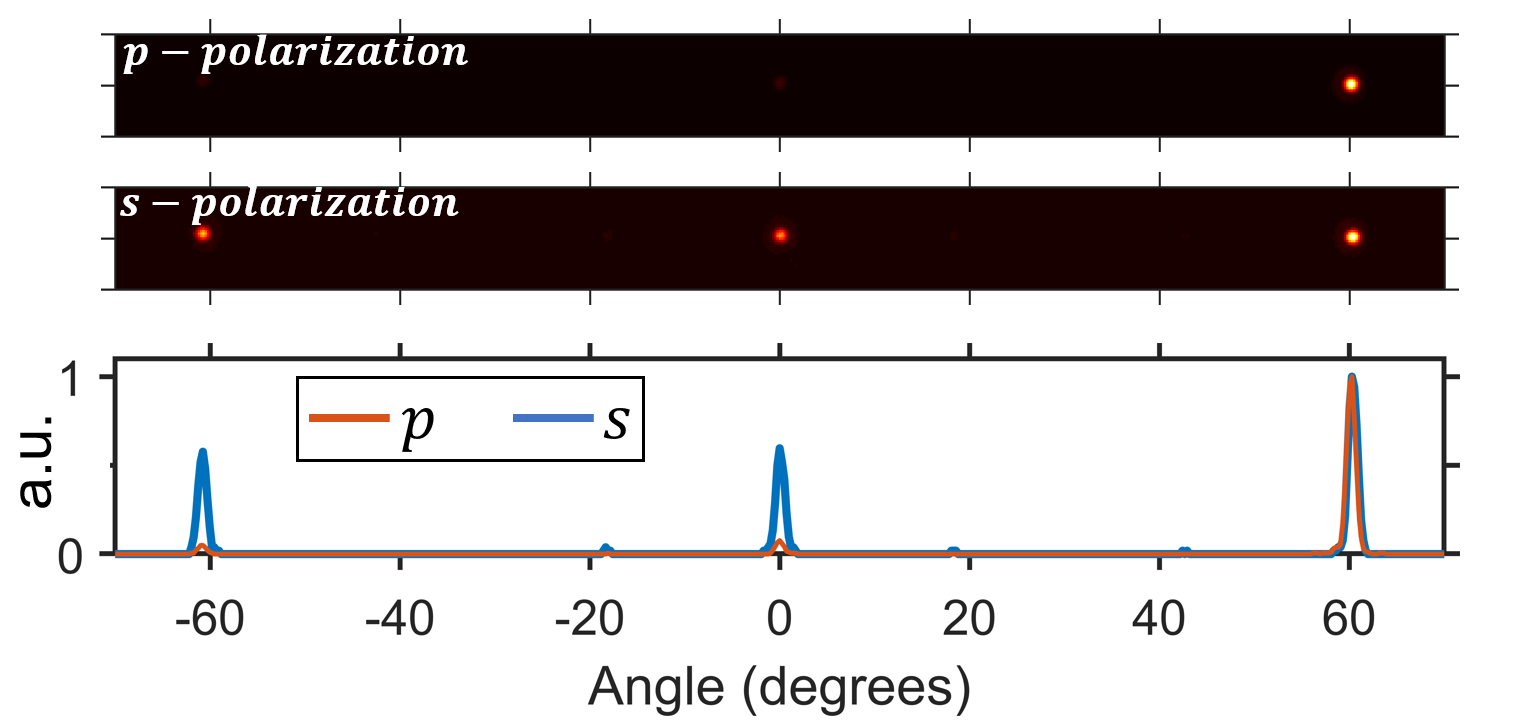


**Figure S2. Measured diffraction efficiencies in transmission.** The diffraction efficiencies of large area (~0.1x0.1 mm^2^) metagratings were recorded in transmission using Fourier imaging (Figure S1). For the optimized structure, the overall transmission coefficients for p- and s-polarization were found to be 70% and 44%, respectively. The power diffraction efficiencies into the three allowed transmission orders were $T_{p, +1}^{exp}=$ 63 %, $T_{p, 0}^{exp}=$ 3.8 %, and $T_{p, -1}^{exp}=$ 2.7 % for p-polarization and $T_{s, +1}^{exp}=$ 19.3 %, $T_{s, 0}^{exp}=$ 11.4 %, and $T_{s, -1}^{exp}=$ 12.5 % for s-polarization. The experimental data agree reasonably well with FDTD results (overall transmission coefficient 75%, $T_{p, +1}^{FDTD}=$ 69%, $T_{p, 0}^{FDTD}=$ 8.6 %, $T_{s, -1}^{FDTD}=$ 5.8 % for p-polarization, and 70%, $T_{s, +1}^{FDTD}=$ 35.6%, $T_{s, 0}^{FDTD}=$ 15.5 %, and $T_{s, -1}^{exp}=$ 21.4 % for s-polarization).

**
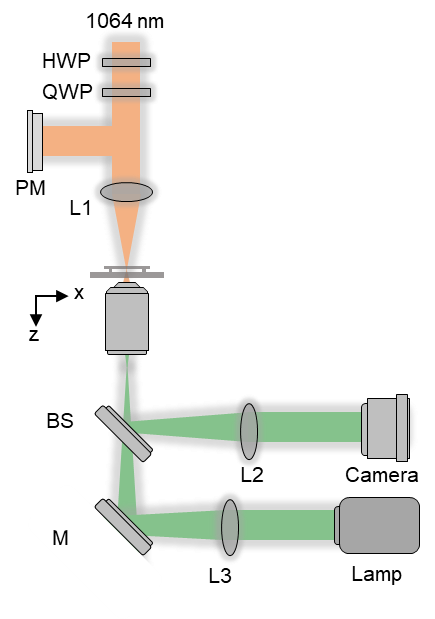
**

**Figure S3. Setup for optical rotation experiments.** Measurements were performed in an inverted microscope (Nikon Eclipse Ti) coupled to a l = 1064 nm CW laser (Cobolt Rumba 2W) using suitable polarization and focusing optics. The metaspinner sample is contained in a thin liquid cell on an automated stage and laser illuminated from above using a *f* = 5 cm lens (L1). The sample is observed in reflection from below using a dry objectives (40X, NA = 0.95 or 20X, NA = 0.7), white-light illumination and a CMOS camera (Thorlabs Zelux). HWP, half-wave plate; QWP, quarter-wave plate; PM, power meter; L, lens; BS, beam splitter; M, mirror.


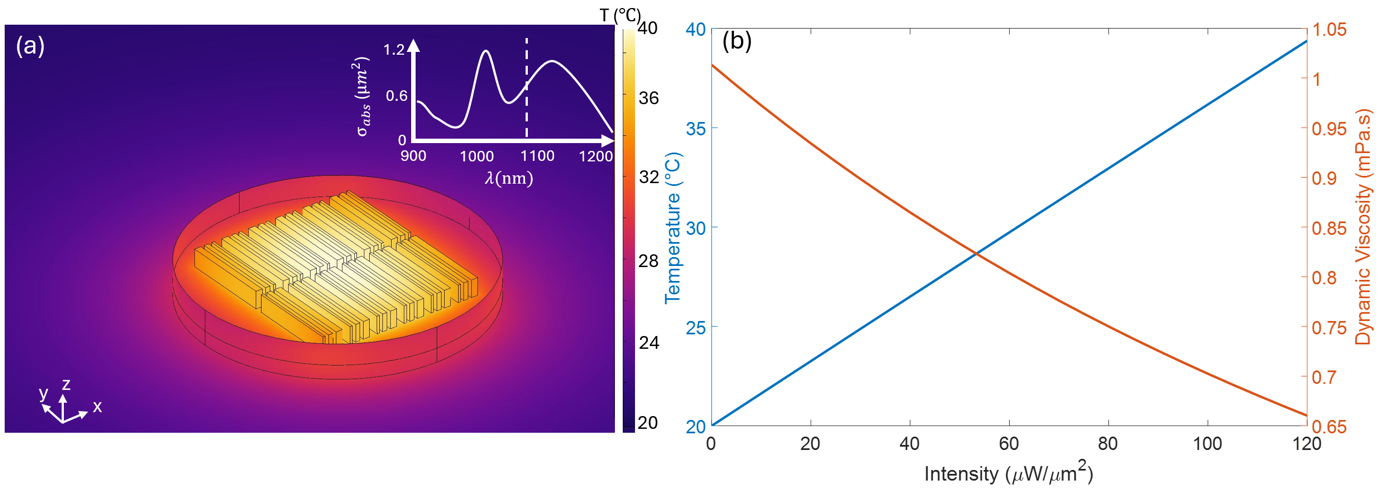


**Figure S4. Finite element simulation of temperature distribution around a metaspinner.** (a) FEM simulation of absolute temperature around a metaspinner illuminated by a 100 μW/μm² s-polarized incident plane wave with wavelength l = 1064 nm. The calculated heat flux density in the metagratings is based on an aSi refractive index of n = 3.8 + i0.0064 at 1064 nm. The inset shows the calculated absorption spectrum of a metagrating unit cell with width 2.5 μm. (b) Simulated maximum temperature around the metaspinner as a function of incident light intensity and the corresponding calculated dynamic viscosity of water.


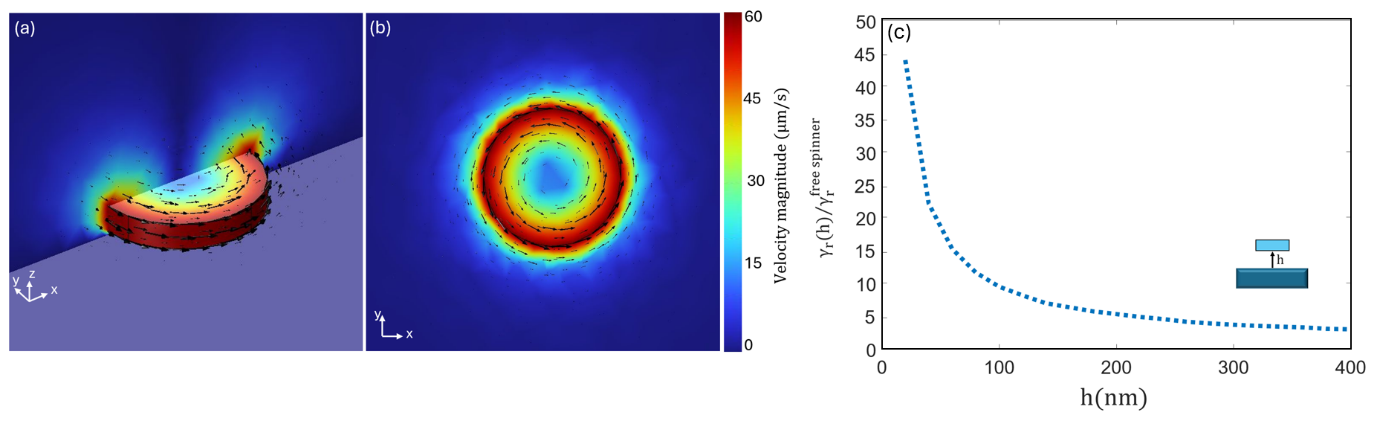


**Figure S5. Fluid dynamics simulations of flow field around a rotating metaspinner.** (a)-(b) simulation of the velocity pattern of water around a metaspinner rotating at an angular velocity of 15 rad/s. The spinner is positioned at a surface-separation of 300 nm from the bottom no-slip boundary of the calculation domain. The upper no-slip boundary is located 50 mm from the bottom, while the domain sidewall boundaries are free, permitting fluid inflow and outflow. The simulations involve two distinct mesh domains: a moving domain containing the spinner and a stationary domain encompassing the rest of the model. A flow continuity condition was applied to the boundary between these domains. c) fluid dynamics simulation of metaspinner rotation drag coefficient $\gamma_{r}\left( h \right)$ versus distance *h* from bottom surface in units of $\gamma_{r}=$ 7.5·10^-19^ Nm·s for a “free” metaspinner located in the middle of the simulation domain.


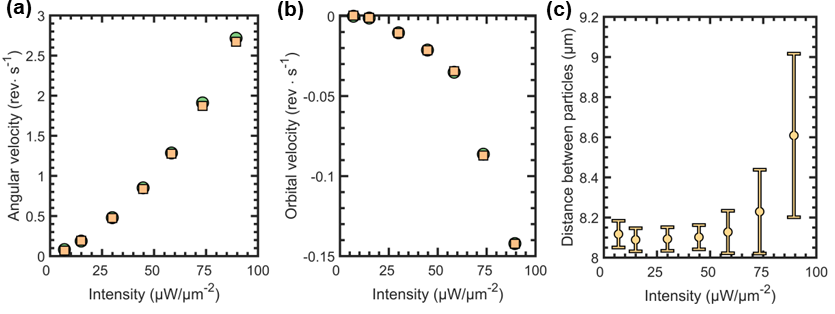


**Figure S6:** **Orbital rotation versus applied intensity.** (a) Spinning frequencies versus intensity for a pair of co-rotating metaspinners. (b) Orbital frequency of the pair versus applied intensity. (c) Estimated center-to-center separation distance between the two metaspinners as a function of applied intensity. The “error-bars” indicate the spread in distances observed over several full trajectories.


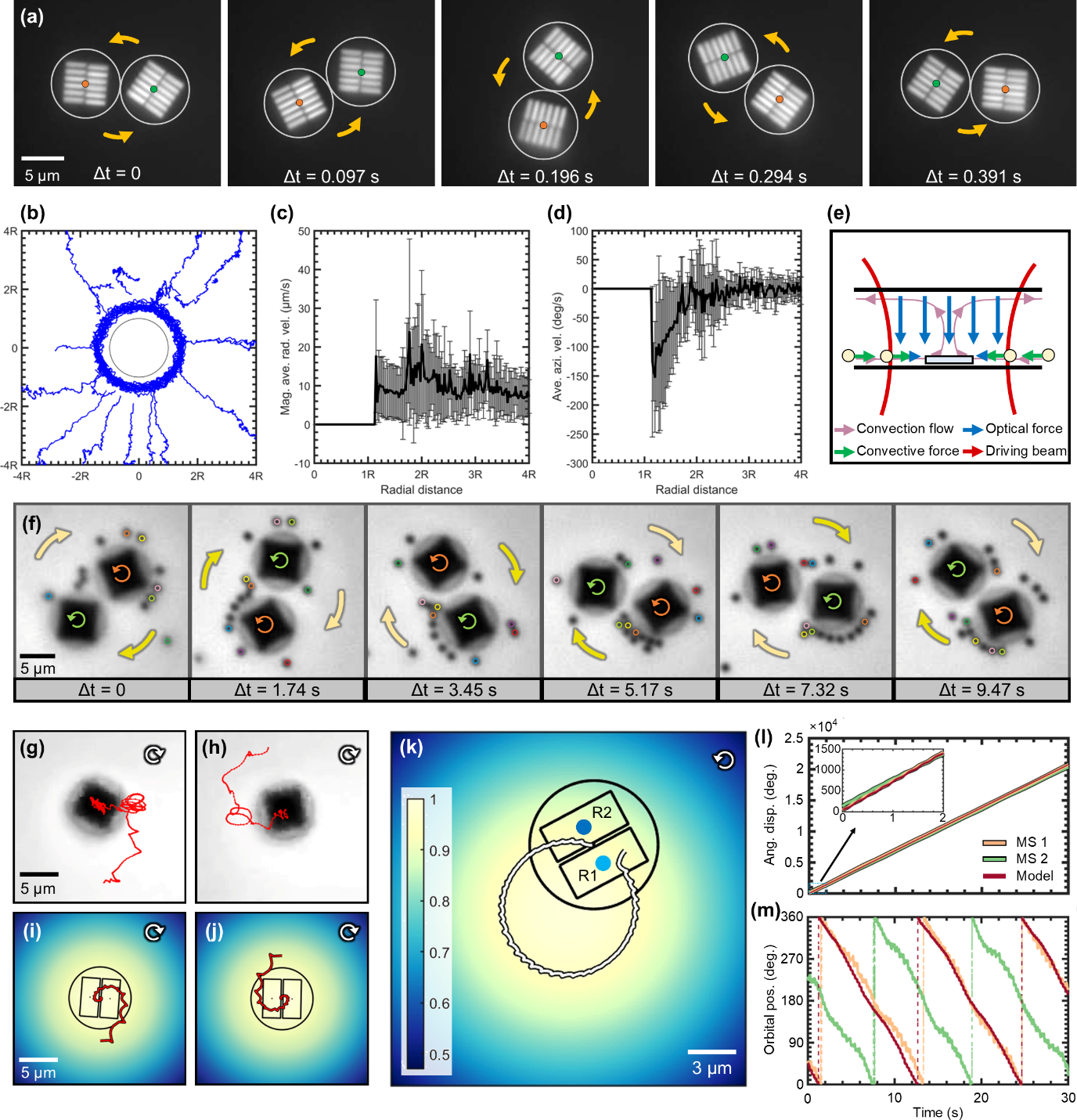


**Figure S7:** **Effect of mechanical locking.** (a) A pair of metaspinners that are initially separate, co-rotating in CCW about their own axes and orbiting in CW, begin to orbit in the opposite direction (CCW) when they accidentally lock together (see also Video S8).

**
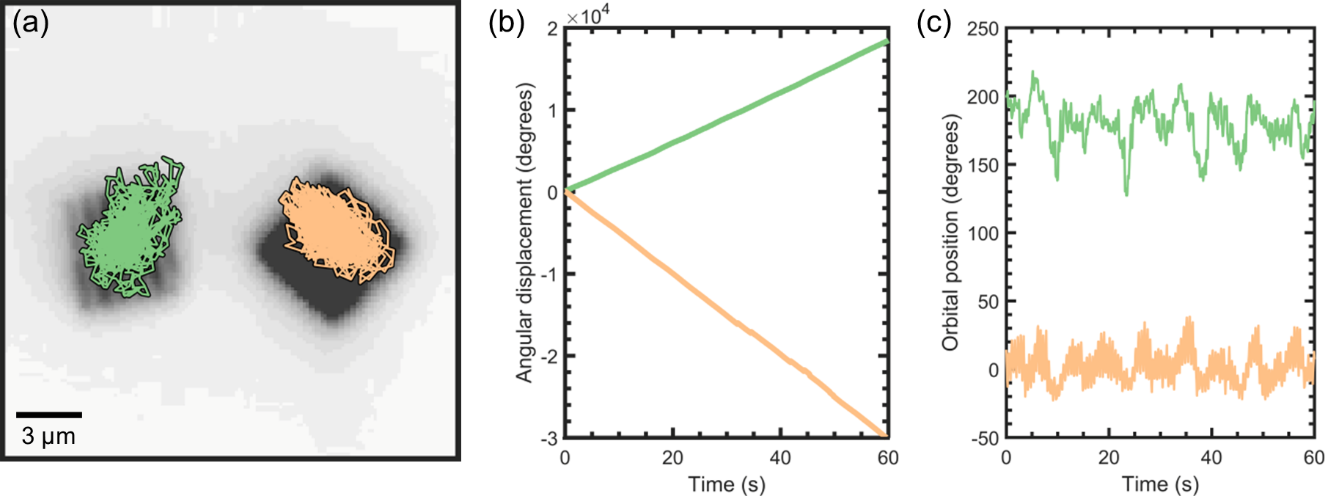
**

**Figure S8**: **Dynamics of a pair of counter-rotating metaspinners.** (a) Tracks of the centroids of a pair of counter-rotating metaspinners subject to a horizontally polarized beam with intensity 60 µW/µm^2^ and the same width as in main Fig. 4. (b) Angular displacement of the metaspinners as they rotate. (c) Orbital position of each metaspinner. The two metaspinners have slightly different spinning frequencies (*f*_1_ ≈ 0.9 Hz, *f*_2_ ≈ 1.4 Hz) but are rotating in opposite directions. The metaspinners still exhibit translational movements consistent with the generated transverse optical gradient force. However, unlike pairs of co-rotating metaspinners, the counter-rotating pair does not exhibit any consistent orbital rotation because of the mutual steric hindrance between the two spinners.


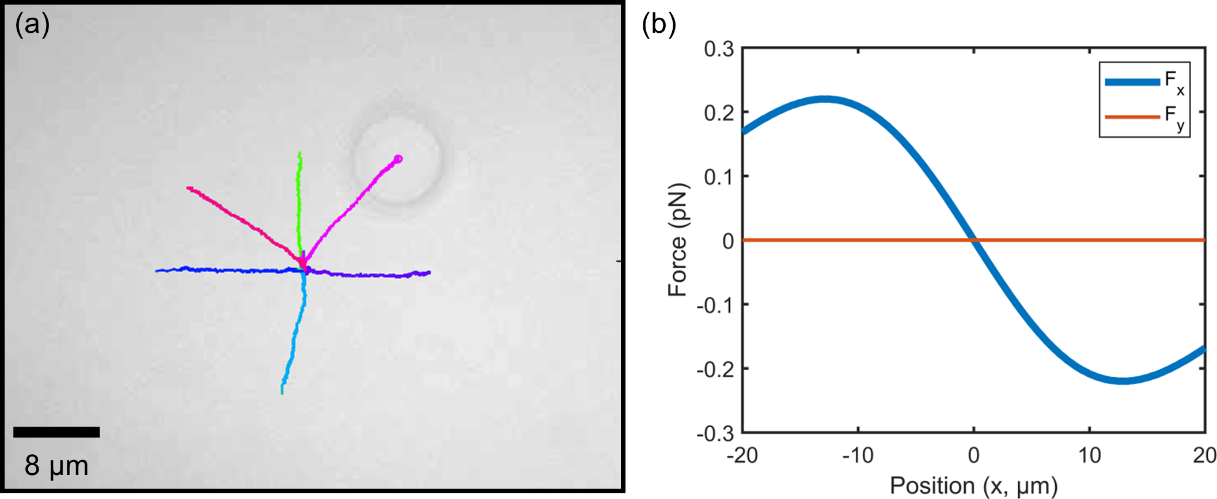


**Figure S9:** **Classical gradient force acting on a SiO_2_ disk.** (a) Tracks of a SiO_2_ disk subject to a x-polarized Gaussian beam with the same width as in main Fig. 4 and intensity 90 µW/µm^2^. The track colors indicate different starting positions. The SiO2 disk has the same dimensions as a metaspinner but it moves radially towards the beam center without any sign of spiral or orbital motion. (b) Lateral force components F_x_ and F_y_ versus SiO_2_ disk displacement from the center of a Gaussian beam with the same characteristics as in a) based on FDTD Maxwell stress tensor analysis.


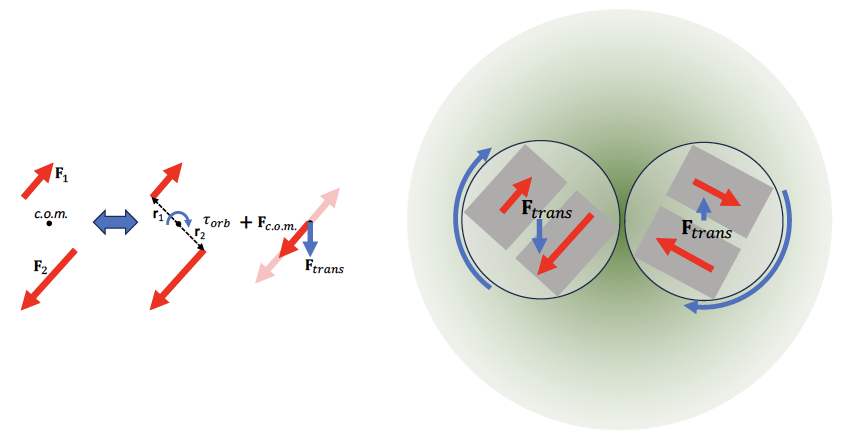


**Figure S10**: **Schematic illustration of optical forces and torques acting on a pair of metaspinners in a Gaussian beam.** The photon recoil forces $\mathbf{F}_{1}$ and $\mathbf{F}_{2}$ acting on the two metagratings in a left-handed (LH) metaspinner situated in a Gaussian intensity gradient result in an orbital torque, $\tau_{orb}$, which spins the particle around its own axis in the clockwise direction, and a force, $\mathbf{F}_{c.o.m.}$, acting on its center of mass. The force component that is transverse to the radial intensity gradient, $\mathbf{F}_{trans}$, causes the spinner to orbit around beam center in the counterclockwise direction, that is, opposite to the spinning motion. Metaspinner movement towards the beam center is driven by the classical optical gradient force (not shown) but blocked through steric hindrance from the other particle in the pair. Hydrodynamic interaction forces work against $\mathbf{F}_{trans}$ but are weak compared to the optical forces.

**Supplementary Videos**

**Video S1.** LH metaspinner rotating in the CW direction. The metaspinner is illuminated by a HP incident beam with peak intensity *I* $\approx$ 75 µW$\cdot$µm^-2^ (actual frame rate).

**Video S2.** RH metaspinner rotating in the CCW direction. The metaspinner is illuminated by a HP incident beam with peak intensity *I* $\approx$ 75 µW$\cdot$µm^-2^ (actual frame rate).

**Video S3.** A single RH metaspinner with tracer particles under HP illumination with peak intensity *I* $\approx$ 90 µW$\cdot$µm^-2^ (actual frame rate).

**Video S4.** Tracer particles under HP illumination with peak intensity *I* $\approx$ 90 µW$\cdot$µm^-2^ (actual frame rate).

**Video S5.** A pair of LH metaspinners co-rotating in the CW direction while mutually orbiting in the CCW direction. The pair is illuminated by a HP incident beam with peak intensity *I* $\approx$ 75 µW$\cdot$µm^-2^ (actual frame rate).

**Video S6.** A pair of RH metaspinners co-rotating in the CCW direction while mutually orbiting in the CW direction. The pair is illuminated by a HP incident beam with peak intensity *I* $\approx$ 75 µW$\cdot$µm^-2^ (actual frame rate).

**Video S7.** a) RH metaspinner dimer with tracer particles under HP illumination with peak intensity *I* $\approx$ 90 µW$\cdot$µm^-2^ (1/3 actual frame rate). b) RH metaspinner dimer with tracer particles under HP illumination with peak intensity *I* $\approx$ 90 µW$\cdot$µm^-2^ (actual frame rate).

**Video S8.** a) A pair of RH metaspinners initially co-rotating in the CCW direction while mutually orbiting in the CW direction, which later get attached to each other and collapse into one unit that rotate in the CCW direction. The pair is illuminated by a HP incident beam with peak intensity *I* $\approx$ 45 µW$\cdot$µm^-2^. b) A collapsed pair of RH metaspinners illuminated by a HP incident beam with peak intensity *I* $\approx$ 90 µW$\cdot$µm^-2^ (actual frame rates).

**Video S9.** A pair of counterrotating metaspinners. The pair is illuminated by a HP incident beam with peak intensity *I* $\approx$ 60 µW$\cdot$µm^-2^ (actual frame rate).

**Video S10.** Dynamic simulation of a RH metaspinner restricted to move within 4.1 µm from the beam center for HP illumination with peak intensity *I* $\approx$ 75 µW$\cdot$µm^-2^ (actual frame rate).

**Videos S11.** Aggregates of *N =* 3-7 co-rotating RH metaspinners. HP illumination with peak intensity *I* $\approx$ 75 µW$\cdot$µm^-2^ for *N* = 3-5 and intensity *I* $\approx$ 30 µW$\cdot$µm^-2^ for *N* = 6 – 7 (actual frame rates).

**Supplementary Text**

In the following, we derive simplified equations that can be used to interpret the experimental results discussed in the main paper. We neglect edge diffraction and assume that the gratings comprising a spinner are irradiated by plane waves incident normal to the grating surface. We only consider forces and torques within the plane of the grating, since this is what we observe experimentally, and we neglect absorption.

**Incident and diffracted fields**

We define a metagrating coordinate system (x’, y’, z’) such that the plane of the grating lies in x’y’-plane and diffraction occurs in the x’z’-plane. The diffraction angles $\theta_{i}$, where $i$ signifies a diffraction order, are measured from the z’-axis and transmitted and reflected orders are treated on an equal footing. The metagrating is situated in a homogeneous medium with refractive index $n$. The torques and transverse forces are obtained from projections on the z’-axis and the x’y’-plane, respectively.

The incident polarization and the forces and torques on the grating are defined/measured in a laboratory frame (x, y, z) with z//z´. We focus on two polarization cases:

1. $\mathbf{E}_{xy}^{0}=E_{0}Re\left\{ \left( \begin{aligned} 1 \\ 0 \end{aligned} \right)e^{i(kz-\omega t)} \right\}$, x-polarized incidence.
2. $\mathbf{E}_{xy}^{0}=\frac{E_{0}}{\sqrt{2}}Re\left\{ \left( \begin{aligned} 1 \\ i \end{aligned} \right)e^{i(kz-\omega t)} \right\}$, LCP incidence.

Here $Re$ means taking the real part, $E_{0}$ is the amplitude of the incident electric field with wavenumber $k$ and angular frequency $\omega$. Assume that the grating axis x’ forms an angle $\varphi$ relative to x. To work out the forces and torques, we need to translate from the grating to the laboratory frame by applying the rotation matrix:

1. $\mathbf{E}_{x'y'}^{0}=\left( \begin{matrix} \cos\left( \varphi\right) & \sin\left( \varphi\right) \\ -sin \left( \varphi\right) & \cos\left( \varphi\right) \end{matrix} \right)\mathbf{E}_{xy}^{0}$

The amplitudes and phases of the diffracted fields are defined through complex 2x2 tensors in planes perpendicular to the propagation directions of the diffracted waves. The tensors will be diagonal since an incident field polarized along x’ will exclusively generate p-polarized diffraction while an incident field polarized along y’ will generate s-polarized diffraction. Diffraction order *i* is thus characterized by the tensor

1. $\left( \begin{matrix} p_{i} & 0 \\ 0 & s_{i}e^{i\psi^{i}} \end{matrix} \right)$

where $p_{i}$ and $s_{i}$ are real numbers (amplitudes) and $\psi^{i}$ is the relative phase shift between the p- and s-polarized components. A metagrating with three transmission and three reflection orders, as is the case considered here, is thus described by 12 amplitudes and 6 relative phases. Multiplying the diffraction tensor with the incident field in the (x’, y’) frame, and omitting $Re$ and the propagation phase for brevity, yields the diffracted fields in the tilted (p_i_, s_i_) frames as:

1. $\mathbf{E}_{ps}^{i}=E_{0}\left( \begin{matrix} p_{i}cos(\varphi) \\ {-s}_{i}e^{i\psi^{i}}sin(\varphi) \end{matrix} \right)$ for x-polarized incidence, and
2. $\mathbf{E}_{ps}^{i}=\frac{E_{0}}{\sqrt{2}}\left( \begin{matrix} p_{i}\left[ \cos\left( \varphi\right)+isin(\varphi) \right] \\ s_{i}e^{i\psi^{i}}\left[ -sin \left( \varphi\right)+icos(\varphi) \right] \end{matrix} \right)$ for LCP incidence.

The corresponding field amplitudes squared are:

1. ${E_{ps}^{i}}^{2}={E_{0}}^{2}\left\{ p_{i}^{2}\cos^{2} (\varphi)+s_{i}^{2}\sin^{2} (\varphi) \right\}$ for x-polarized incidence, and
2. ${E_{ps}^{i}}^{2}=\frac{{E_{0}}^{2}}{2}\left\{ p_{i}^{2}+s_{i}^{2} \right\}$ for LCP incidence.

**Reaction force acting on a single grating.**

The Minkovski linear momentum $\mathcal{p}$ carried by a beam with power $P$ per time unit is

1. $\frac{\partial\mathcal{p}}{\partial t}=n\frac{P}{c_{0}}$

where $n$ is the refractive index. By projecting the diffracted momenta on the x’-axis, through multiplication with $sin(\theta_{i})$, correcting for the change in wave-front area from the incident to the diffracted beams through multiplication with $\left| cos(\theta_{i}) \right|$, to ensure energy conservation, we obtain the net reaction force in the x’-direction by summing over all diffraction orders and taking the negative. For x-polarized incidence, we get:

1. $F_{x^{'}}=-\frac{n}{c_{0}}P_{0}\sum_{i} \left[ p_{i}^{2}\cos^{2} \left( \varphi\right)+s_{i}^{2}\sin^{2} \left( \varphi\right) \right]\cdot sin{(\theta}_{i})\cdot\left| cos(\theta_{i}) \right|=-\frac{n}{c_{0}}P_{0}\sum_{i} \left[ T_{p,i}\cos^{2} \left( \varphi\right)+T_{s,i}\sin^{2} \left( \varphi\right) \right]\cdot sin{(\theta}_{i})$

while for LCP, we get:

1. $F_{x^{'}}=-\frac{n}{{2c}_{0}}P_{0}\sum_{i} \left[ p_{i}^{2} +s_{i}^{2} \right]\cdot sin{(\theta}_{i})\cdot\left| cos(\theta_{i}) \right|=-\frac{n}{{2c}_{0}}P_{0}\sum_{i} \left[ T_{p,i}+T_{s,i} \right]\cdot sin{(\theta}_{i})$

We have here utilized that the incident power $P_{0}$ that hits the grating is given by the incident irradiance (intensity) $I_{0}$ times the grating area $A$ as $P_{0}=I_{0}A$, while the irradiance is given by the incident electric field amplitude $E_{0}$ as $I_{0}=\frac{\epsilon_{0}c}{2}E_{0}^{2}$, and we have introduced power transmission/reflection coefficients $T_{p,i}=p_{i}^{2}\left| cos(\theta_{i}) \right|$ and $T_{s,i}=s_{i}^{2}\left| cos(\theta_{i}) \right|$ for the different polarizations and diffraction orders.

Finally, since there is no force in the y’-direction, the force components in the (x,y) system simply becomes:

1. $\mathbf{F}_{\boldsymbol{xy}}=\left[ \begin{matrix} {cos(\varphi)F}_{x^{'}} \\ {sin(\varphi)F}_{x^{'}} \end{matrix} \right]$

**Spin torque acting on a single grating.**

The spin density of a plane wave is directed parallel to the propagation direction and given by:

1. $\left\langle\mathbf{s} \right\rangle={Im\left[ {\epsilon_{0}\mathbf{E}}^{*}\times\mathbf{E} \right]}/{2\omega}$

By inserting the incident and diffracted fields above, we can calculate the spin densities for the two polarization cases considered. After some algebra, one obtains for x-polarized incidence:

1. $\left\langle\mathbf{s}_{0} \right\rangle=0$
2. $\left\langle\mathbf{s}^{\boldsymbol{i}} \right\rangle=-\hat{k}_{i}\frac{\epsilon_{0}E_{0}^{2}}{2\omega}p_{i}s_{i}\sin\left( \psi_{i} \right)\sin\left( 2\varphi\right)$

while LCP incidence gives:

1. $\left\langle\mathbf{s}_{0} \right\rangle=\hat{z}\frac{\epsilon_{0}E_{0}^{2}}{2\omega}$
2. $\left\langle\mathbf{s}^{\boldsymbol{i}} \right\rangle=\hat{k}_{i}\frac{\epsilon_{0}E_{0}^{2}}{2\omega}p_{i}s_{i}cos(\psi_{i})$

where $\hat{k}_{i}$ indicates the direction of propagation of the diffracted waves.

We can now write down the torque exerted on a grating with area $A$ by following the same procedure as for the linear momentum transfer and forces in the xy-plane:

1. $\tau_{\hat{z}}=\frac{c_{0}}{n}A\left\langle\hat{z}\cdot\mathbf{s}_{0} \right\rangle-\frac{c_{0}}{n}A\sum_{i} \left\langle\hat{k}_{i}\cdot\mathbf{s}^{\boldsymbol{i}} \right\rangle\cos\left( \theta_{i} \right)\left| cos(\theta_{i}) \right|$

where the first $\cos\left( \theta_{i} \right)$ factors project out the actual $\hat{z}$-components of the diffracted torque. Writing out the full expression for the torques in terms of power, we obtain for x-polarized incidence:

1. $\tau_{\hat{z}}=\frac{1}{n\omega}P_{0}\sin\left( 2\varphi\right)\sum_{i} p_{i}s_{i}\sin\left( \psi_{i} \right)\cos\left( \theta_{i} \right)\left| cos(\theta_{i}) \right|$

while for LCP, we find:

1. $\tau_{\hat{z}}=-\frac{1}{n\omega}P_{0}\left\{ 1-\sum_{i} p_{i}s_{i}\cos\left( \psi_{i} \right)\cos\left( \theta_{i} \right)\left| cos(\theta_{i}) \right| \right\}$

The torque obtained for x-polarization can be thought of as an “alignment torque” since it strives to orient the grating parallel to the incident polarization due to the $\sin\left( 2\varphi\right)$ factor. The magnitude of the alignment torque is maximum for $\varphi=$ ±45^o^ and ±135^o^ and it vanishes when the grating is either aligned with or perpendicular to the polarization. The ”spin torque” obtained for LCP incidence is, in contrast, independent of grating orientation.

**Torques acting on a metaspinner**


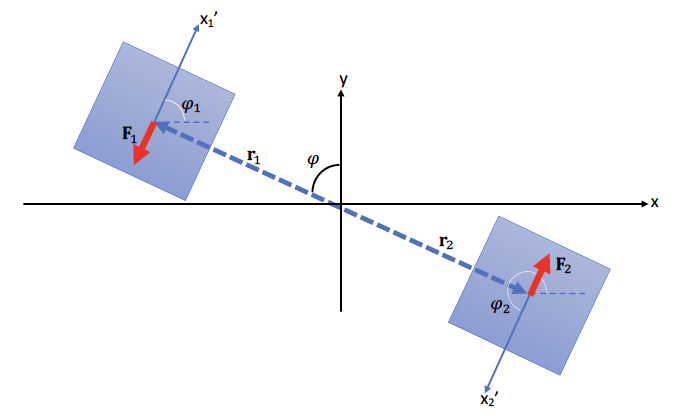


We describe a metaspinner as two gratings connected by a rigid bar, as in the figure above. The gratings are oriented such that the xy-plane projections of their main diffraction orders are perpendicular to the bar and points in opposite directions. We can then add the torques from each grating to obtain the total torque on the spinner. The torques originating in the translation forces are classified as “orbital torque” while the torques derived from polarization conversion are classified as “spin torque”. The figure above illustrates a right-handed (RH) metaspinner, designed to rotate in the counterclockwise (CCW) direction. For this case, we have

$$\varphi_{1}=\varphi, \varphi_{2}=\varphi+\pi$$

1. $\mathbf{r}_{1}=r\left[ \begin{matrix} -sin(\varphi) \\ cos(\varphi) \end{matrix} \right], \mathbf{r}_{2}=r\left[ \begin{matrix} -sin(\varphi+\pi) \\ cos(\varphi+\pi) \end{matrix} \right]=-\mathbf{r}_{1}$

$\mathbf{F}_{1}=F_{x'}\left[ \begin{matrix} cos(\varphi) \\ sin(\varphi) \end{matrix} \right], \mathbf{F}_{2}=F_{x'}\left[ \begin{matrix} cos(\varphi+\pi) \\ sin(\varphi+\pi) \end{matrix} \right]=-\mathbf{F}_{1}$

We then obtain (note that $P_{0}$ signifies the power incident on one of the gratings in the spinner only):

Orbital torque, x-polarization:

1. $\tau_{orb}\hat{z}=\mathbf{r}_{1}\times\mathbf{F}_{1}+\mathbf{r}_{2}\times\mathbf{F}_{2}=\hat{z}2r\frac{n}{c_{0}}P_{0}\sum_{i} \left( p_{i}^{2}\cos^{2} (\varphi)+s_{i}^{2}\sin^{2} (\varphi) \right)sin{(\theta}_{i})\left| cos(\theta_{i}) \right|=\hat{z}2r\frac{n}{c_{0}}P_{0}\sum_{i} \left( T_{p,i}\cos^{2} (\varphi)+T_{s,i}\sin^{2} (\varphi) \right)sin{(\theta}_{i})$

Orbital torque, LCP:

1. $\tau_{orb}\hat{z}=\mathbf{r}_{1}\times\mathbf{F}_{1}+\mathbf{r}_{2}\times\mathbf{F}_{2}=\hat{z}r\frac{n}{c_{0}}P_{0}\sum_{i} \left( T_{p,i} +T_{s,i} \right)sin{(\theta}_{i})$

Spin torque, x-polarization:

1. $\tau_{spin}\hat{z}=\tau_{spin}^{1}\hat{z}+\tau_{spin}^{2}\hat{z}=\hat{z}\frac{2}{n\omega}P_{0}\sin\left( 2\varphi\right)\sum_{i} p_{i}s_{i}sin(\psi_{i})\cos\left( \theta_{i} \right)\left| cos(\theta_{i}) \right|$

Spin torque, LCP

1. $\tau_{spin}\hat{z}=\tau_{spin}^{1}\hat{z}+\tau_{spin}^{2}\hat{z}=-\hat{z}\frac{2}{n\omega}P_{0}\left\{ 1-\sum_{i} p_{i}s_{i}cos(\psi_{i})\cos\left( \theta_{i} \right)\left| cos(\theta_{i}) \right| \right\}$

The total torque is given by the sum of the orbital and spin contributions:

1. $\tau_{\hat{z}}=\tau_{orb}+\tau_{spin}$

It is also useful to calculate the torque magnitudes averaged over one full rotation cycle, $\varphi=0\to2\pi$:

1. $\frac{1}{2\pi}\int_{0}^{2\pi} \tau_{orb}^{x-pol} (\varphi)d\varphi=\frac{1}{2\pi}\int_{0}^{2\pi} \tau_{orb}^{LCP} (\varphi)d\varphi=r\frac{n}{c_{0}}P_{0}\sum_{i} \left( T_{p,i} +T_{s,i} \right)sin{(\theta}_{i})$
2. $\frac{1}{2\pi}\int_{0}^{2\pi} \tau_{spin}^{x-pol} (\varphi)d\varphi=0$
3. $\frac{1}{2\pi}\int_{0}^{2\pi} \tau_{spin}^{LCP} (\varphi)d\varphi=-\frac{2}{n\omega}P_{0}\left\{ 1-\sum_{i} p_{i}s_{i}cos(\psi_{i})\cos\left( \theta_{i} \right)\left| cos(\theta_{i}) \right| \right\}$

**Forces and torques acting on a metaspinner in a unidirectional intensity gradient**

We consider the same RH metaspinner as before, but now it is situated in a field $I\left( x \right)$ that slowly varies in intensity along *x*. We approximate the intensity that hits the two metagratings with the intensity at their respective center positions $\mathbf{r}_{1}$ and $\mathbf{r}_{2}$. The positions along the x-axis are given by the instantaneous angles, $\varphi_{1}=\varphi$ and $\varphi_{2}=\varphi_{1}+\pi$, and the moment arm $r$, which yields:

1. $I\left( x_{1} \right)=I\left( x_{c.o.m} \right)-{r\cdot\sin\left( \varphi\right)\frac{dI}{dx}|}_{x_{c.o.m}}, I\left( x_{2} \right)=I\left( x_{c.o.m} \right)+{r\cdot\sin\left( \varphi\right)\frac{dI}{dx}|}_{x_{c.o.m}}$

where $x_{c.o.m}$ is the *x*-position of the metaspinner center of mass (c.o.m.). The net force acting on the c.o.m. is given by the vectorial sum of the two translation forces, i.e. $\mathbf{F}_{c.o.m.}=\mathbf{F}_{1}+\mathbf{F}_{2}$. The forces are in turn proportional to the metagrating area $A$ and the local intensities. We then have for the LCP case:

1. $\mathbf{F}_{c.o.m.}=\mathbf{F}_{1}+\mathbf{F}_{2}=-I\left( x_{1} \right)AC_{LCP}\left[ \begin{matrix} \cos\left( \varphi\right) \\ \sin\left( \varphi\right) \end{matrix} \right]-I\left( x_{2} \right)AC_{LCP}\left[ \begin{matrix} \cos\left( \varphi+\pi\right) \\ \sin\left( \varphi+\pi\right) \end{matrix} \right]$

where:

1. $C_{LCP}=\frac{n}{{2c}_{0}}\sum_{i} \left( T_{p,i} +T_{s,i} \right)sin{(\theta}_{i})$

Inserting the intensities yields:

1. $\mathbf{F}_{c.o.m.}=2AC_{LCP}r\sin\left( \varphi\right)\left[ \begin{matrix} \cos\left( \varphi\right) \\ \sin\left( \varphi\right) \end{matrix} \right]{\frac{dI}{dx}|}_{x_{c.o.m}}=AC_{LCP}r\left\{ \left[ \begin{matrix} 0 \\ 1 \end{matrix} \right]+\left[ \begin{matrix} \sin\left( 2\varphi\right) \\ -cos \left( 2\varphi\right) \end{matrix} \right] \right\}{\frac{dI}{dx}|}_{x_{c.o.m}}$

The intensity gradient in the x-direction thus gives rise to two distinct contributions to the force, where the first is independent of orientation $\varphi$ and always points perpendicular to the intensity gradient. We term this component the *transverse gradient force*, $\mathbf{F}_{trans}$. For the RH metaspinner and ${dI}/{dx>0}$, $\mathbf{F}_{trans}$ points in the positive y-direction while a LH metaspinner would experience a force in the opposite direction. The direction of $\mathbf{F}_{trans}$ is thus determined by the handedness of the spinner and, thereby, by its spinning direction. As illustrated in Fig. S11, the result is that a metaspinner situated at some fixed radial distance $R_{c.o.m.}>r$ from the center of a Gaussian beam will orbit the beam center in the opposite direction to its spinning motion. $\mathbf{F}_{trans}$ will, in this special case, manifest as a fictious *gradient orbital torque* with magnitude $AC_{LCP}rR_{c.o.m.}{\frac{dI}{dx}|}_{R_{c.o.m.}}$.

The second force component in Eq. (33) also has constant magnitude, but it varies with orientation $\varphi$ such that a circular movement of the c.o.m. is induced. However, it does not result in a net displacement if averaged over all angles. The net result of the two force components is thus that the metaspinner will move in a spiral pattern in the direction perpendicular to the intensity gradient.

In addition to $\mathbf{F}_{c.o.m.}$ above, the metaspinner is subject to the classical gradient force, $\mathbf{F}_{grad}$, which always points in the direction of the intensity gradient, here along *x*. $\mathbf{F}_{grad}$ does not appear in the derivation above, however, and has to be added ad hoc.

We can use Eq. (33) also for x-polarized incidence, but we need to replace $C_{LCP}$ with

1. $C_{x-pol}=\frac{n}{c_{0}}\sum_{i} \left( T_{p,i}\cos^{2} (\varphi)+T_{s,i}\sin^{2} (\varphi) \right)sin{(\theta}_{i})$

The magnitude of $\mathbf{F}_{trans}$ will now fluctuate with $\varphi$, but it will remain positive since $C_{x-pol}$ does not change sign as $\varphi$ varies. Similarly, the angular variation of $C_{x-pol}$ will in general cause the second force component in Eq. (33) to drive an elliptical rather than a circular motion.

The net torques relative to the metaspinner c.o.m., located at $\mathbf{r}_{c.o.m.}$, can be obtained in a similar fashion as above, but they do not depend on the intensity gradient. The results are thus simply:

Orbital torque, x-polarization:

1. $\tau_{orb}\hat{z}=\mathbf{(r}_{1}-\mathbf{r}_{c.o.m.})\times\mathbf{F}_{1}+\mathbf{(r}_{2}-\mathbf{r}_{c.o.m.})\times\mathbf{F}_{2}=\hat{z}2rI\left( x_{c.o.m} \right)AC_{x-pol}$

Orbital torque, LCP:

1. $\tau_{orb}\hat{z}=\mathbf{(r}_{1}-\mathbf{r}_{c.o.m.})\times\mathbf{F}_{1}+\mathbf{(r}_{2}-\mathbf{r}_{c.o.m.})\times\mathbf{F}_{2}=\hat{z}2rI\left( x_{c.o.m} \right)AC_{LCP}$

Spin torque, x-polarization:

1. $\tau_{spin}\hat{z}=\tau_{spin,1}\hat{z}+\tau_{spin,2}\hat{z}=\hat{z}\frac{2}{n\omega}I\left( x_{c.o.m} \right)A\sin\left( 2\varphi\right)\sum_{i} p_{i}s_{i}sin(\psi_{i})\cos\left( \theta_{i} \right)\left| cos(\theta_{i}) \right|$

Spin torque, LCP

1. $\tau_{spin}\hat{z}=\tau_{spin,1}\hat{z}+\tau_{spin,2}\hat{z}=-\hat{z}\frac{2}{n\omega}I\left( x_{c.o.m} \right)A\left\{ 1-\sum_{i} p_{i}s_{i}cos(\psi_{i})\cos\left( \theta_{i} \right)\left| cos(\theta_{i}) \right| \right\}$
